# The immune response after fracture trauma is different in old compared to young patients

**DOI:** 10.1186/s12979-014-0020-x

**Published:** 2014-12-11

**Authors:** Helen Vester, Markus S Huber-Lang, Qerim Kida, Alexander Scola, Martijn van Griensven, Florian Gebhard, Andreas K Nüssler, Mario Perl

**Affiliations:** Department of Trauma Surgery, Klinikum rechts der Isar, Technical University Munich, Munich, Germany; Department of Orthopedic Trauma, Hand-, Plastic-, and Reconstructive Surgery, University Hospital Ulm, Ulm, Germany; Siegfried Weller Institute for Trauma Research, BG Trauma Center Tübingen, Eberhard Karls University Tübingen, Tübingen, Germany; BG-Trauma Center Murnau, Prof.-Küntscher-Str. 8, 82418 Murnau, Germany

**Keywords:** Immunoageing, Trauma, Apoptosis, Inflammation

## Abstract

**Background:**

Despite significant medical progress and improved treatment, surgical procedures of proximal femur fractures in older patients are still associated with a high postoperative complication and mortality rate. Recently, several authors investigated the phenomenon of immunoageing, indicating differences in the ageing immune system.

The aim of the present multi-center prospective clinical trial was to analyze differences in the posttraumatic immune response of old patients compared to young patients.

**Methods:**

Blood was collected from young patients (<50 y, n = 20) with long bone fractures (YF), old patients (>70 y, n = 21) with proximal femur fractures (OF) upon clinical admission and within 6 hours after surgery, and two healthy age matched control groups (YH & OH). Serum TRAIL- and cytokine concentrations were analyzed via cytometric bead array, Fas-Ligand and TNF-Receptor-I via ELISA. CD15^+^ magnetic bead-isolated neutrophils (PMN) were TUNEL stained.

**Results:**

IL-6 was significantly increased only in OF after trauma and surgery whereas YF patient exhibited a marked decrease of TNF after trauma. Interestingly, a significant increase of GM-CSF serum levels was observed in YF only, whereas OF exhibited a decrease of systemic IFN-γ concentrations after trauma and after surgery. The healthy controls, old and young, had more or less similar inflammation levels.

Moreover, TRAIL serum levels were diminished in OF after trauma and even further after surgery whereas in YF this was only observed after the surgical procedure. Fas-L concentrations were reduced only in YF after surgery or trauma. PMN apoptosis was significantly reduced only in YF, indicating activation of the innate immune system.

**Discussion:**

In summary, our data suggest that the posttraumatic immune response is differently regulated in old and young trauma patients. The operative procedure further impacts these differences after trauma. Whether the decreased activation of PMNs and phagocytes along with the observed dysregulation of the posttraumatic inflammatory response contributes to the high perioperative mortality rate of the elderly suffering from a proximal femoral fracture requires further investigation.

## Background

Hip fractures are among the most common traumatic injuries in the old patient [1,2]. Due to the overageing of our society, the incidence increases and is expected to be doubled by the year 2050. Despite improving (peri-) operative management, the post-operative complication rate (26%), the in-house mortality rate (6%) and the one-year mortality rate (30%) still remain fairly high [3,4]. Post-operative complications like pneumonia, wound-infections, urinary tract infections, etc. also result in delayed recovery and prolonged in-hospital stay. The overall outcome as well as the quality of life are negatively affected and cause high costs for the health care system [5,6].

Previous studies indicated changes in the immune system function of the elderly, indicating a malfunction, which could be responsible for the high susceptibility to infections post operatively. The ageing of the immune system is referred to as “immunosenescence”. Ageing per se is associated with increased levels of circulating inflammatory components in the blood including TNF-α and IL-6, as well as of the anti-inflammatory IL-1RA even in the absence of trauma [5,7-9].

Moreover, increased apoptosis has been reported in the aged immune system while the development of apoptosis resistance is also discussed as a key mechanism of immunosenescence. For example, some investigators have reported a decrease of Fas-mediated apoptosis in aged animals [10,11].

TNF-α, a central mediator of apoptosis induced cell death, is markedly increased in the elderly [12]. Recent reports [13] also showed an increased susceptibility to TNF-α-induced apoptosis in lymphocytes from aged humans.

A well balanced apoptosis of immune cells is eminent for proper cell cycle and healthy ageing [14].

The inflammatory response after trauma has been extensively studied and SIRS, CARS and MARS have been indicated to exists in a variety of settings of patients following trauma [15,16]. However, the impact of a traumatic insult on the aged immune system is poorly understood. So far, little data exist concerning posttraumatic inflammation and apoptosis in old patients. Regarding high postoperative complication rates of the old patient, there might be a malfunction of the immune system or an impaired apoptosis rate of immune cells. Knowledge of the specific posttraumatic regulation/dysregulation of the immune response in the old patients could help improving posttraumatic therapy strategies and treatment concepts specifically designed for this patient population.

## Results

### Patients

A total of 93 patients and volunteers were enrolled: 21 old patients suffering from hip fracture and 20 young patients suffering from a fracture of a long bone as well as 26 young healthy volunteers, 26 old healthy controls. For details see Table 1.

**Table 1:** **Enrolled patients and controls**

**Group**	**Number**	**Age (MW ± SD) [years]**	**Gender ratio male:female**
Young healthy control	26	29 ± 3	15:11
Old healthy control	26	78 ± 6	15:11
Young with fracture	20	31 ± 9	12:8
Old with fracture	21	86 ± 7	7:14

A total of 41 patients and 52 controls could be included.

### Inflammation: cytokine profile

**Figure 1:**
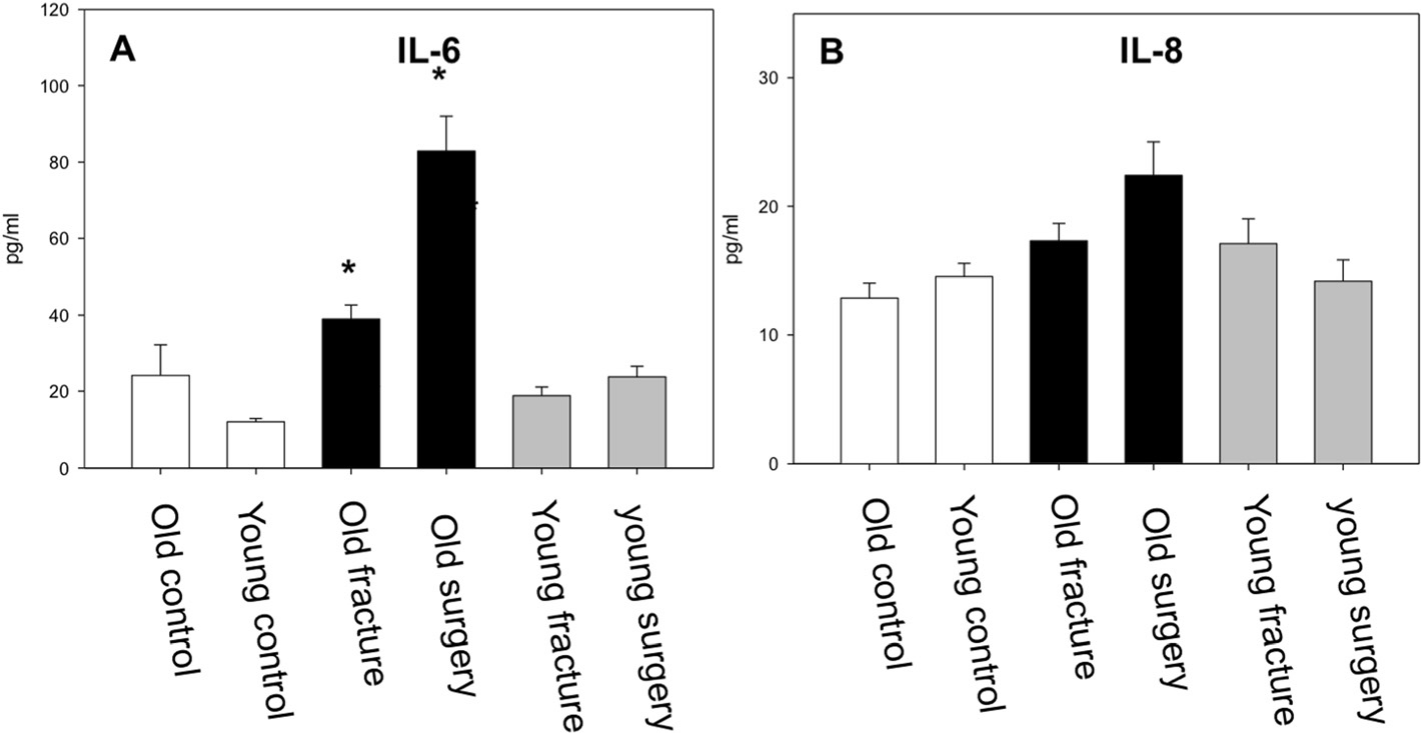
**IL-6 and IL-8 levels. A** While the young and old controls showed nearly similar IL-6 levels, trauma and surgery led to a significant increase in old patients compared to controls while serum levels in the young patients did not change markedly (p < 0.05, ANOVA on Ranks). **B** No significant differences in IL-8 levels could be detected.

IL-6 serum concentrations were significantly increased in old patients with fracture upon admission and even more after surgery when compared to old controls. In contrast, IL-6 levels in young patients were not increased by the traumatic insult or operative procedure. No significant differences were found in serum IL-6 levels between old and young healthy volunteers (Figure 1A).

Changes of IL-8 serum levels upon admission and after surgery showed a trend similar to IL-6, but differences did not reach statistical significance (Figure 1B).

**Figure 2:**
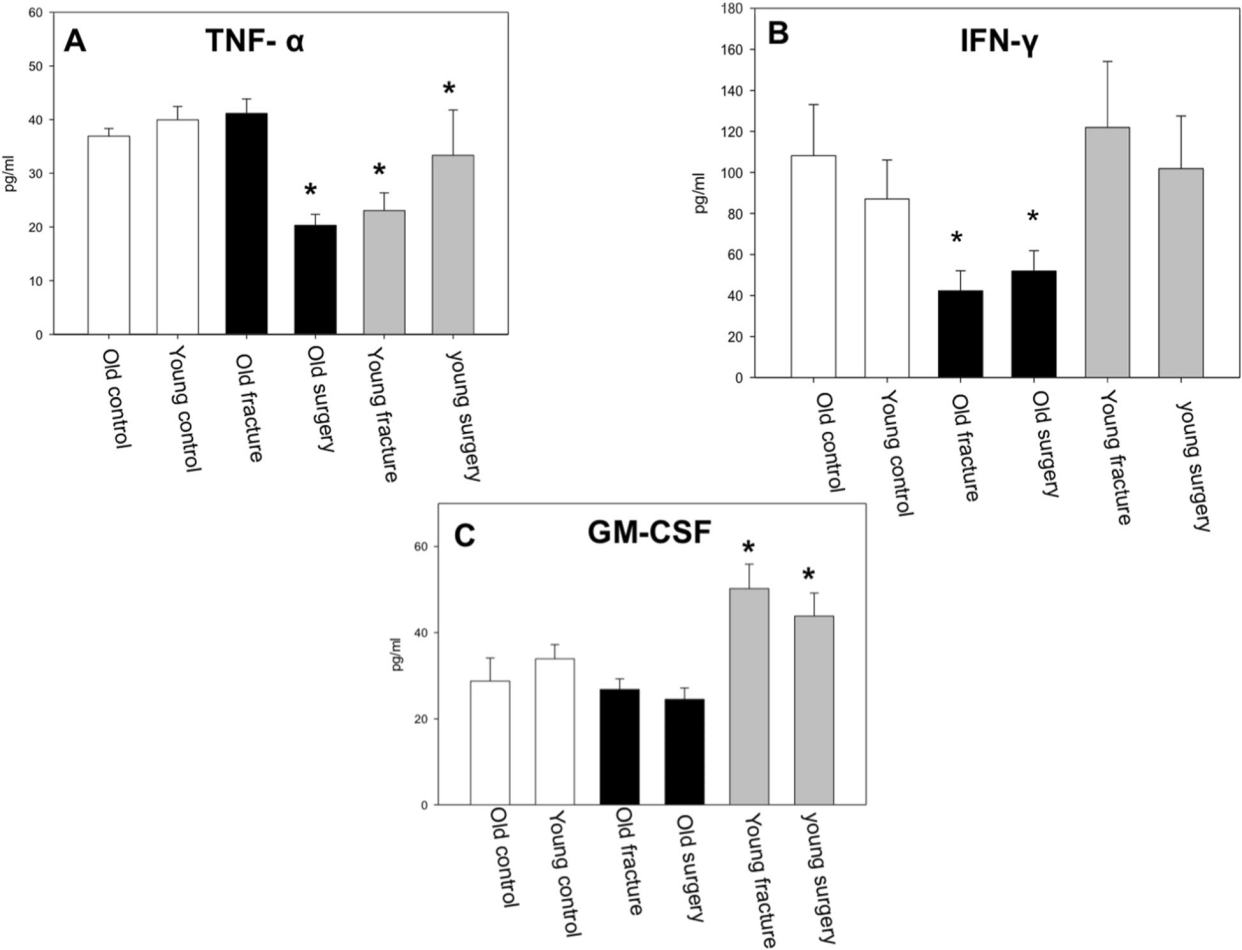
**TNF-α, IFN-γ and GM-CSF levels. A** While TNF-α was significantly decreased in young patients after trauma and surgery, the old patients showed a reduction only after surgery compared to the healthy control respectively (p < 0.05, ANOVA on ranks). **B** In contrast IFN-γ concentrations were significantly reduced in old patients after trauma and surgery while no changes could be detected in young patients compared to healthy controls respectively (p < 0.05, Anova on ranks). **C** Young patients experienced an immediate increase of GM-CSF levels after trauma. This was not observed in old patients (p < 0.05, ANOVA on ranks).

In old patients TNF-α serum concentrations were not altered in response to trauma, however, surgery after trauma led to a significant decrease of TNF- α levels (Figure 2A). Following trauma as well as after the surgical procedure serum TNF-α concentrations were markedly decreased in young patients when compared to their healthy controls (Figure 2A).

Furthermore INF-γ serum levels were significantly reduced in the old patients suffering from hip fracture after trauma and after surgery compared to the control. On the contrary, INF-γ levels in the young patients with fracture of a long bone remained at the base level after trauma and surgery (Figure 2B).

Only young patients with fracture showed a significant increase of GM-CSF serum levels after trauma and surgery whereas no changes were detected in old patients with hip fracture (Figure 2C).

No significant differences were observed in serum concentrations of IL-10, IL-1β and IL-12 (data not shown).

### Apoptosis

**Figure 3:**
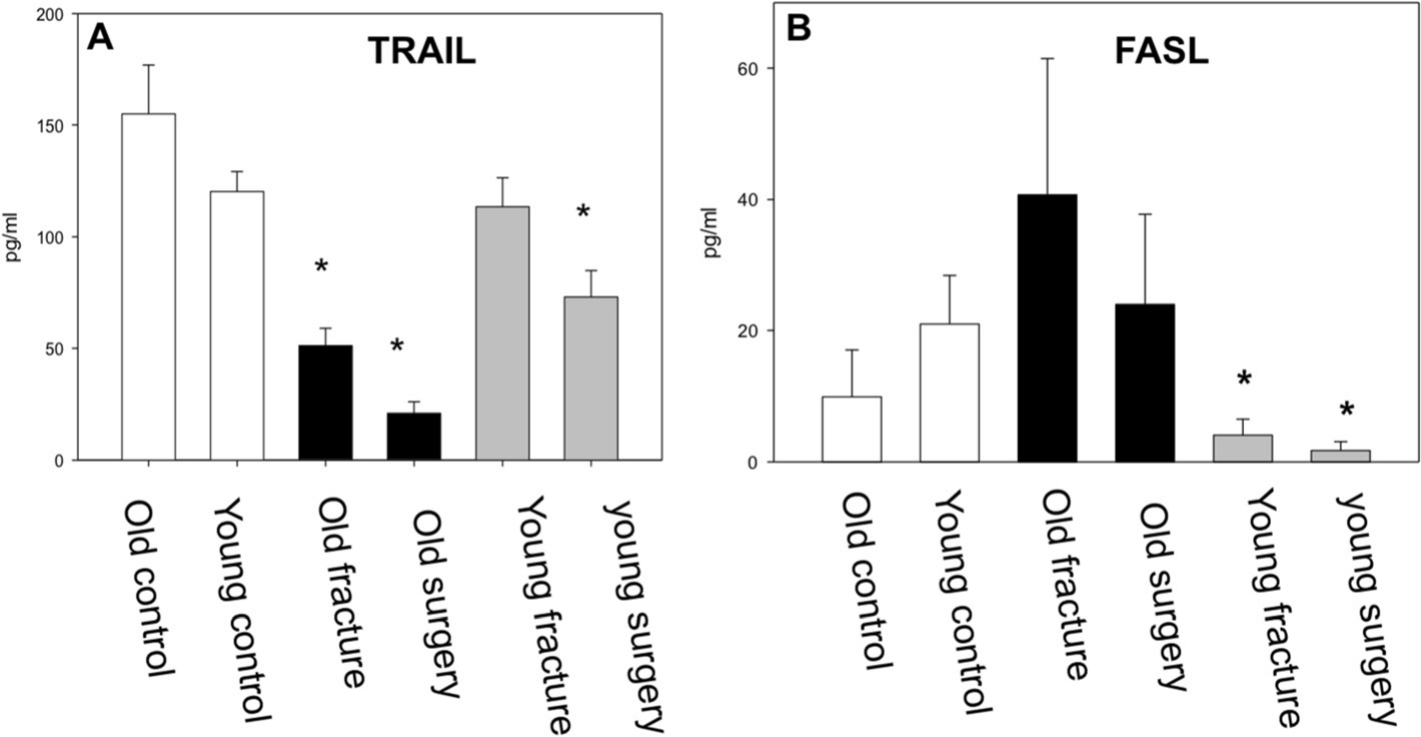
**Apoptosis. A** TRAIL expression was immediately significantly reduced in the old patients compared to the healthy control. The reduction in the young patients was significant after surgery but not as distinct as seen in the old patients (p < 0.05, ANOVA on ranks). **B** FasL was significantly reduced in the young patients after trauma and surgery while no changes could be detected in the old patients (p < 0.05, Anova on ranks).

The apoptosis inducing TRAIL-receptor was significantly reduced after trauma as well as following surgery in old trauma patients. Interestingly, in young patients trauma alone did not but the following operative procedure led to a marked decrease of TRAIL receptor serum levels (Figure 3A).

Serum Fas-Ligand concentrations were substantially reduced in young patients following trauma and also after surgery when compared to healthy controls. In old patients, a tendency towards higher FasL levels after trauma was observed but interindividual variability was high, therefore a statistical significance was not observed (Figure 3B).

**Figure 4:**
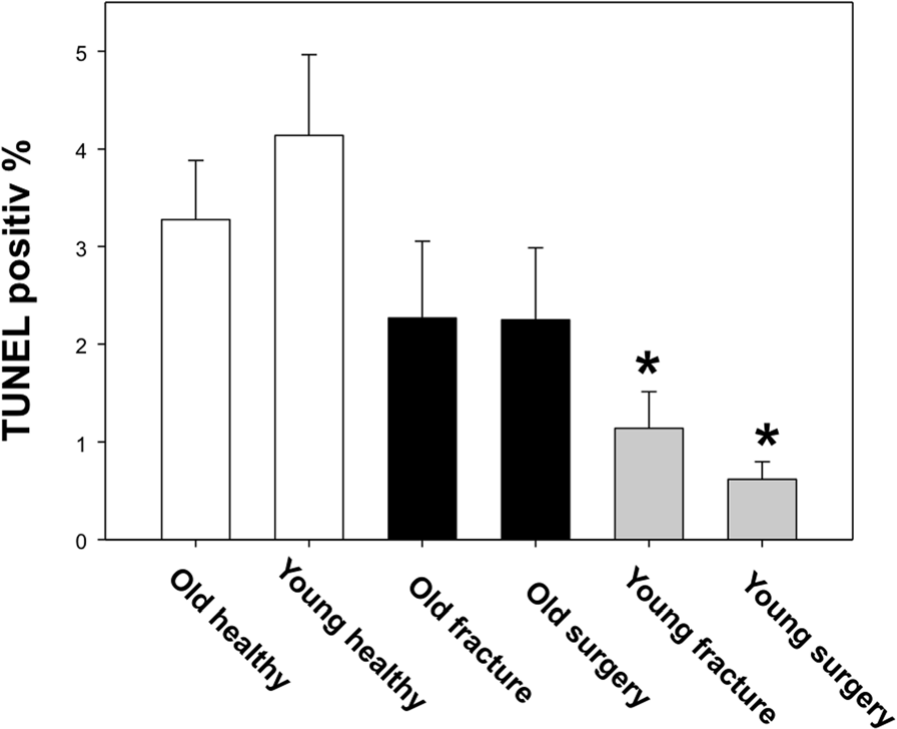
**Neutrophil Apoptosis.** Granulocyte apoptosis in the young patients was significantly decreased after trauma and surgery while no changes were detected in old patients compared to healthy controls (p < 0.05, ANOVA on ranks).

Interestingly, substantial differences in the apoptotic response of circulating PMN between old and young patients were noticed. Here, only young patients’ PMN responded to trauma and surgery with a significant decrease of TUNEL positive PMNs, indicating posttraumatic activation of neutrophils. In old trauma patients no such response was detected (Figure 4).

## Discussion

Elderly patients are more susceptible to postoperative complications and they respond differently to trauma compared to young patients suffering from similar injuries. Due to the overageing of our society the phenomenon of the so-called *immunoageing* becomes more and more of public interest. So far, only little data exist concerning the immune system and the immune response of the aged patient after trauma. Here, we have investigated in a prospective clinical double center trial whether the systemic inflammatory and the apoptotic response of circulating neutrophils are different in old and young patients following trauma and subsequent surgery. Our data indicates, that there are substantial differences between these two groups.

The minimum age of 70 for the old patients was chosen because other studies indicated significant changes in the immune system of the elderly people above the age of 65 [17-19]. Quian et al. for example showed reduced expression of Toll-like receptor 1 (TLR1) in PMNs in older (>65 years) adults. Stimulation through TLR1 led to lower activation of integrins (CD11b and CD18), lower production of the chemokine IL-8, and lower levels of the phosphorylated signaling intermediate p38 MAP kinase when compared to PMN from younger donors (21–30 years). [17] Plonquet et al. described an increased susceptibility for nosocomial infections in the elderly patient (>70 years), which was related to an ageing of the immune system [19].

In our study, older patients showed a significant increase of IL-6 concentrations after trauma and surgery whereas young patients did not respond to these insults. Both young healthy and old healthy controls showed similar baseline levels of IL-6. IL-6 is a prognostic marker for trauma outcome and development of multiple organ failure. Increased posttraumatic IL-6 levels have been associated with unfavorable outcome [20,21]. In this regard, Pape et al. indicated that IL-6 levels >800 pg/ml were associated with increased organ failure in multiple injured patients [22]. A similar trend was observed for IL-8. In this regard, particularly IL-6 has been suggested to be a reliable indicator for trauma severity and outcome [15,16]. Furthermore, Pape and colleagues indicated, that even after major trauma, when IL-6 is already elevated, additional major surgery further increased systemic IL-6 levels indicating that IL-6 could be a reliable parameter to assess the traumatic and surgical load to an individual [23]. In our study, old patients reached highest levels after surgery around 80 pg/ml. According to previous studies [16] these levels are similar to patients with multiple injuries and an ISS of up to 17 early after trauma, thus reflecting a considerable inflammatory response of the immune system. Thus, our data indicate that in elderly patients an isolated fracture of the femur elicits an inflammatory response similar to a low injury severity in polytrauma patients. Moreover young patients in our study did not respond with an increase of IL-6 concentrations after an isolated fracture of the lower extremity, indicating that such a traumatic insult was not sufficient to trigger a significant posttraumatic response at the time points investigated.

While other authors showed increased IL-6 and TNF-α levels in the healthy elderly person [24,25] we could not detect statistically significant differences in our healthy control groups, indicating that the increased IL-6 levels in old patients seen here were truly induced by the traumatic or surgical insult.

In contrast, fractures led to a significant decrease of TNF-α concentrations in the young patient population, while trauma itself did not change TNF-α levels in the old patient. Surgery led to a significant decrease of serum TNF-α levels in the old patient. In this regard, TNF-α has been described to increase early after trauma, but because it peaks early TNF-α release can easily be missed.

One reason for the increased incidence of infectious disease, accompanied by increased morbidity and mortality in the elderly might be a reduced capacity to produce IFN-γ. This cytokine plays an important role in defense against intracellular pathogens such as mycobacteria and viruses. Several authors showed a significant decrease in IFN-γ production in vitro after stimulation with bacterial products (LPS) or viral antigens (influenza vaccine) [26].

These results are supported by our findings, which indicated a significant decrease of IFN-γ concentrations in old patients after trauma and surgery. In contrast, systemic IFN-γ levels in young patients after trauma and surgery remained unchanged when compared to controls. Whether reduced IFN-γ serum levels after trauma are associated with high postoperative complication rates in the old patient still needs to be determined.

Kim et al. showed significant differences in cytokine profiles in healthy young and old people [27]. They compared the profiles of 22 cytokines, including chemokines and growth factors, in a case-controlled study group of a gender-matched, healthy cohort of 55 patients over the age of 65 and 55 patients under the age of 45. They detected significant lower levels of G-CSF, granulocyte-monocyte colony-stimulating factor (GM-CSF), and monocyte chemoattractant protein-1 (MCP-1) in the elderly. Moreover, partial correlation analysis demonstrating the correlation between cytokine levels when controlled for gender, systolic blood pressure, total cholesterol, HDL cholesterol, triglyceride, and serum creatinine levels further demonstrated that G-CSF, GM-CSF, and MCP-1 had significant negative correlations with age.

In our study, young patients responded after trauma and surgery with a marked increase of serum GM-CSF concentrations whereas GM-CSF levels remained unchanged in the old patients when compared to controls. GM-CSF (and also G-CSF) has been indicated to play an important role in leukocyte development from hematopoietic stem cells. It also appears to be an important mediator of the host response to infection [28]. In this regard, recruitment of monocytes and neutrophils, the key components of the first line of defense, to local tissue sites in response to infection or inflammation is triggered by GM-CSF. Moreover, it regulates other cell types in addition to neutrophils and monocytes such as natural killer cells and dendritic cells [29]. Lendemann et al. indicated in a prospective clinical experimental study with six polytraumatized patients that all showed reduced cytokine production and HLA-DR expression on monocytes. Administration of GM-CSF in vitro significantly increased the level of HLA-DR expression on monocytes and GM-CSF application increased IL-10-levels after LPS-stimulation [30]. GM-CSF is primarily released by monocytes, macrophages and lymphocytes after stimulation by cytokines like IL-1 or TNF. In this regard, the lack of response of a systemic release of GM-CSF in old trauma patients here might reflect a dysregulation of this cell type in the older population.

Polymorphonuclear granulocyte apoptosis was significantly reduced in the young patients after trauma and surgery while no changes in the old patients could be detected. In this regard, delayed PMN apoptosis after trauma or sepsis has been interpreted as part of PMN activation preparing to ward off a potential or ongoing infection [31]. PMNs are one of the first cells to arrive at the site of infection, where they can directly eliminate pathogens. Their activation and recruitment into peripheral tissues is an important step to ward off infection. With ageing, alterations of the PMN activation and recruitment are described [32]. Thus, Wenisch et al. showed an impaired neutrophil bactericidal activity as well as an impaired neutrophil chemotaxis with increasing age of the neutrophils [33]. However, posttraumatic activation of PMN can also be harmful, as activated PMN in the absence of invading pathogens are able to potentiate tissue damage. In this regard, we have previously demonstrated, that further delaying neutrophil apoptosis after hemorrhage via myeloid overexpression of BCL-2 increases survival after a septic infection but not a septic inflammatory challenge [34].

An age-related impairment of Fas/FasL mediated apoptosis is believed to contribute to compromised regulation of the immune system and immunosenescence [11]. Kavathia et al. could show a decrease in apoptotic markers (cytochrome c) and proapoptotic factors (sFasL) and an increase in anti-apoptotic factors in circulation with increasing age [35]. The age related shift in favor of reduced apoptosis may contribute to reduced clearance of immune cells leading to a state of chronic inflammation [36]. Our data showed a significant decrease of Fas-Ligand in young patients, while it was increased in the old patient after trauma compared to the healthy old control. This suggests an age dependent different activation of apoptosis pathway. In this regard, a decrease in circulating Fas-Ligand levels after trauma may indicate increased binding of FasL to the CD95-receptor on cell membranes indicating increased apoptosis in young patients [37].

Interestingly, our data also indicate that TRAIL serum levels were diminished in old patients after trauma and even more so after surgery while in young trauma patients only surgery but not trauma alone led to a marked reduction of TRAIL serum concentrations. In this regard, TRAIL plays an important role in the modulation of inflammatory responses, especially in the process of immune paralysis. Tian et al. could show that low plasma TRAIL levels were associated with immune paralysis and a high risk of mortality in patients with septic shock. They concluded that TRAIL could present a potential biomarker of immune function and predict the survival of septic patients [38].

## Conclusion

Inflammation and regulation of apoptosis after trauma are important processes that allow the injured individual to prepare to ward off potential invading pathogens. However, these processes are also involved in mediating organ damage. Here we have found substantial differences in the inflammatory and apoptotic response following fracture trauma between young and old patients, indicating malregulation of the immune system after trauma in old patients. Whether the observed differences are directly associated with the increased complication rate and susceptibility to infections in old patients requires additional studies.

This study has several limitations. Although we excluded patients suffering from chronic diseases or cancer, the patient collective was inhomogeneous. In this age group, healthy patients without any medication or former medical history are extremely rare. Furthermore because trauma cannot be anticipated and baseline sample collection in trauma patients before the incident is not possible our control groups consist of different individuals when compared to our trauma group, yet no differences in age or gender between these groups were detected.

### Patients and methods

#### Ethics statement

Blood sampling was conducted in accordance with the Declaration of Helsinki (1964) and its amendments. The study protocol was approved by the hospital’s Ethics Committee (reference numbers 2676/10 Munich Rechts der Isar, 24/10 University of Ulm, Surgical Center Ulm) and informed consent was obtained from all subjects. Patients with chronic diseases, cancer or who were suffering from open fractures were excluded as well as pregnant or underage patients or patients who took immune modulating medicaments like corticosteroids were not included in the study.

#### Patients

Patients were recruited at two level I university trauma centers (RDI, UU). Two different age groups were studied: Young patients aged less than 50 years and old patients aged over 70 years. In both age groups, we distinguished between healthy controls and patients with a fracture of the long bones. In both groups of patients, the blood sampling was performed on admission as well as within 6 h after surgery. Blood samples from the control patients were taken once.

#### Granulocyte isolation

45 ml venous blood (36 ml EDTA (ethylenediaminetetraacetic acid) blood and 9 ml serum blood) was collected from each patient at the two time points indicated above. The granulocytes were isolated out of the EDTA blood by MACS using CD15 magnetic microbeads according to the manufacturer’s instructions (Miltenyi Biotec, Cologne, Germany) as described before previously [39]. Cell purity was over 95% as assessed by FACS analysis.

#### TUNEL staining

After selection of CD15 positive Granulocytes, TUNEL staining, as described previously, was performed following the manufacturer’s instructions (In situ cell death detection kit; Roche, Basel, Switzerland) [40].

#### Multiplex assay and ELISA

In the human samples, serum levels of IL6, IL-8, GM-CSF, TRAIL, TNF-α and IFN-γ were measured using the cytometric bead array technique (BD™ Cytometric Bead Array Inflammation Kit, BD Biosciences) according to the manufacturers descriptions and in the technique as described previously [31]. According to the manufacurer’s manual, it is possible to detect as low as 0.274 pg/mL of the relevant protein with this multiplex-assay. FasL and TNF-RI were measured via ELISA (R&D Systems, Wiesbaden-Nordenstadt, Germany) according to the manufactures instructions.

#### Statistical analysis

Results are presented as mean ± SEM. A one-way analysis of variance and a one-way analysis of variance on ranks followed by Student-Newman-Keuls and Dunn test, respectively, as a post hoc test for multiple comparisons were performed to determine significant differences between experimental means. A P value of <0.05 was considered statistically significant.

## Abbreviations

TRAIL: Tumor necrosis factor related apoptosis inducing ligand-receptor
FASL: Fas ligand
IL: Interleukin
PMN: Polymorphonuclear granulocyte
TNF: Tumor necrosis factor
GM-CSF: Granulocyte-monocyte colony-stimulating factor
IFN: Interferon
SIRS: Systemic inflammatory response syndrome
CARS: Compensatory antiinflammatory response syndrome
MARS: Mixed antagonistic response syndrome
TUNEL: TdT-mediated dUTP-biotin nick end labeling
TLR1: Toll-like receptor 1
ISS: Injury severity score
